# Optimizing Growth: The Case for Iodine

**DOI:** 10.3390/nu15040814

**Published:** 2023-02-05

**Authors:** Jessica Rigutto-Farebrother

**Affiliations:** 1 Laboratory of Nutrition and Metabolic Epigenetics, Institute of Food, Nutrition and Health, ETH Zürich, LFV E 14.1, Schmelzbergstrasse 7, CH-8092 Zürich, Switzerland; jessica.rigutto@hest.ethz.ch; 2 Global Center for the Development of the Whole Child, University of Notre Dame, 200 Visitation Hall, Notre Dame, IN 46556, USA

**Keywords:** iodine, growth, iodine deficiency, thyroid function

## Abstract

Iodine is an essential micronutrient and component of thyroid hormone. An adequate dietary iodine intake is critical to maintain and promote normal growth and development, especially during vulnerable life stages such as pregnancy and early infancy. The role of iodine in cognitive development is supported by numerous interventional and observational studies, and when iodine intake is too low, somatic growth is also impaired. This can be clearly seen in cases of untreated congenital hypothyroidism related to severe iodine deficiency, which is characterized, in part, by a short stature. Nevertheless, the impact of a less severe iodine deficiency on growth, whether in utero or postnatal, is unclear. Robust studies examining the relationship between iodine and growth are rarely feasible, including the aspect of examining the effect of a single micronutrient on a process that is reliant on multiple nutrients for optimal success. Conversely, excessive iodine intake can affect thyroid function and the secretion of optimal thyroid hormone levels; however, whether this affects growth has not been examined. This narrative review outlines the mechanisms by which iodine contributes to the growth process from conception onwards, supported by evidence from human studies. It emphasizes the need for adequate iodine public health policies and their robust monitoring and surveillance, to ensure coverage for all population groups, particularly those at life stages vulnerable for growth. Finally, it summarizes the other micronutrients important to consider alongside iodine when seeking to assess the impact of iodine on somatic growth.

## 1. Introduction

Growth is a multi-factorial process. Adequate macro- and micronutrient intake, socioeconomic status, environmental and sanitary conditions, as well as culture, all impact on the risk of sub-optimal growth in a population [1,2,3,4]. Growth faltering in early life, if uncorrected, may compromise height in adulthood leading to permanent stunting. Stunting has far-reaching adverse consequences, including poor educational performance, lost productivity and low wages as an adult [3]. Though perhaps often more associated with cognition through its implication in the development of the brain, iodine is also a critical micronutrient for somatic growth. It exerts its effects via thyroid hormone (TH), being an integral and essential component. TH regulates vital processes in the body from the agglomeration of fetal cells after conception [1] through to the maintenance of cellular energy and metabolism in adulthood [1], and the role of iodine in skeletal formation and development via thyroid hormone is known [5]. However, inadequate iodine intakes may result in low serum TH concentrations.

Iodine deficiency disorders (IDD; Table 1) are well described and can be as severe as congenital hypothyroidism and miscarriage in pregnancy [6]. Growth can also be impaired: in particular, stunting is marked in myxedematous congenital hypothyroidism caused by severe iodine deficiency during gestation [4,7]. However, the precise effect of iodine on growth trajectories in individuals subject to a lesser degree of iodine deficiency is not as clear.

This is partly because somatic growth is difficult to adequately assess in nutrition intervention studies. Not only because growth is so multifactorial, as outlined above, thereby making it difficult to assess the effect in relation to a single micronutrient, but moreover, because growth is uneven: at times of peak development such as infancy and childhood, growth phases may be rapid, and individuals develop at different rates. Yet, for the purposes of clinical research, growth is slow. Studies looking to measure a difference in somatic growth in response to a given intervention will require a sufficiently long period between baseline and endpoint as well as a large sample size to permit the estimation of group differences with a reasonable certainty [8]. Randomized controlled trials (RCT) are not always feasible for ethical reasons, and interpretations from cross-sectional studies are limited, since, by definition, they cannot describe the effect of an intervention and may be subject to bias [9]. Therefore, indicators other than height and, for example, height-for-age Z-scores, to indicate changes in the growth trajectory of an individual, must be considered.

This narrative review presents the physiological mechanisms by which iodine contributes to growth, including for both insufficient and excessive iodine intakes, and presents evidence from human studies that demonstrate these possible effects.

## 2. Thyroid Function and Growth

### 2.1. Iodine as a Component of Thyroid Hormone

TH synthesis is stimulated by the secretion of thyrotropin-releasing hormone (TRH) from the hypothalamus, which in turn stimulates the anterior pituitary gland to synthesize and secrete thyrotropin, or thyroid-stimulating hormone (TSH). TSH acts directly on the thyroid gland to stimulate the synthesis of TH, completing the so-called hypothalamus–pituitary–thyroid (HPT) axis [10].

One of the principal steps in the synthesis of TH is the organification of iodine, that is, the iodination of tyrosine residues on the structure of the prohormone thyroglobulin, present in the colloid of thyroid follicular cells. The coupling of these residues forms triiodothyronine (T3) and thyroxine (T4), which are secreted from the thyroid cell following release from thyroglobulin. Circulating TH is usually bound to carrier proteins, including thyroxine-binding globulin, albumin and transthyretin [5]. Only approximately 0.2% of T3 and 0.02% of T4 are unbound in plasma [5] and it is these free, unbound hormones that act on the hypothalamus and anterior pituitary gland to inhibit the synthesis of further TH, in a negative feedback loop that regulates the HPT axis. Thus, TH status is maintained in a normal range for that individual.

The only known functional role of iodine in the body is for the formation of TH, and it is required at physiological doses from conception throughout life (Table 2), being particularly critical periconceptually and during pregnancy and early infancy, as described later. Iodine enters the circulation as plasma inorganic iodide, I^−^, and is cleared principally by the thyroid gland for hormone production, and kidney for excretion in urine. A healthy euthyroid adult would have approximately 15–20 mg iodine, of which 70–80% would be stored in the thyroid [11], and thyroidal iodine content has been shown to be positively correlated with TH concentrations [12]. However, thyroidal iodine stores will decrease when exposed to inadequate intakes or if not continually repleted during certain life stages, for example, in pregnancy, where the maternal iodine supply must account for maternal and fetal needs [13,14,15,16].

The principal sources of dietary iodine are seaweeds and seafoods [21], animal milk, particularly in countries where herds are fed with iodine-fortified feed or where the dairy industry permits iodine-based disinfectants [22,23], eggs [21], and iodized salt. Salt iodization is considered a public health success; increasing global efforts to iodize salt for human and animal consumption has led to the near elimination of previously widespread iodine deficiency leaving few countries still at risk [24]. It is a highly cost-effective intervention, at only USD 0.06 per capita per year or less [25], and reaches all population groups [26].

### 2.2. Thyroid Hormone and Growth

TH acts on almost every cell in the body, except for the adult brain and spleen. The key periods for growth across the life cycle—pregnancy, early infancy and lactation, childhood—are therefore crucial for ensuring optimal iodine intakes. During skeletal development and linear bone growth, bone mass accumulates and bone mineralization increases until peak bone mass is achieved, usually by early adulthood [5].

Growth hormone (GH) and the insulin-like growth factors (IGFs) promote growth through action on the organs associated with key metabolic effects including the liver, skeletal muscle, and bone [27]. Through the somatropic GH-IGF axis, GH promotes the synthesis and secretion of IGFs, which are proteins typically produced in the liver in response to GH stimulation [28]. IGFs, in turn, enhance cell proliferation and differentiation [29]. GH also stimulates the production of different growth factors (epidermal growth factor, nerve growth factor, and erythropoietin).

The physiological mechanisms linking iodine and growth arise through the important and intricate links between TH, GH, and the IGFs. TH influences the expression and action of GH [30,31], and the secretion of GH is dependent upon normal thyroid function [32,33]. Further, the synthesis of TH requires the presence of IGF-1 [34], and IGF-1 itself is necessary for the anabolic effect of T3 [33]. Additionally, GH accelerates the peripheral conversion of T4 to T3 [35]. During childhood, beginning from between six months and three years to puberty, the GH-IGF-1 axis and TH become more influential on growth, and at puberty, GH and IGF-1 concentrations increase significantly in both males and females [36].

Yet, some of the effects of TH on growth are direct and occur independently of the effects of GH. These include effects on bone and skeletal development [37,38].This has been modelled in mice, where Kim and Mohan proposed that the increase in IGF-1 expression and bone growth during prepubertal and pubertal periods are mediated via TH- and GH-dependent mechanisms, respectively [37].

The skeleton is a target for TH action, and TH receptors have been identified in osteoblast cell lines and human bone samples [39], though the effect of TH on osteoclasts may be, rather, indirect [39]. Biochemical studies have shown that TH affects the expression of various bone markers in serum that reflect changes in both resorption and formation [39]. As in the thyroid gland itself, a delicate balance in bone exists between the inactive hormone T4 and its active metabolite, T3. This balance is facilitated by the D2 iodothyronine deiodinase enzyme in osteoblasts, and the inactivating iodothyronine deiodinase D3, which inactivates both T4 and T3 with the removal of a 5-iodine atom. D3 is present in all skeletal cell lines during development and until weaning [40,41]. It is thought that D3 is essential in limiting TH availability to the immature skeleton [41].

### 2.3. Mechanisms by which Suboptimal Iodine Intakes May Affect Growth

#### 2.3.1. Iodine Deficiency and Hypothyroidism

Hypothyroidism is the inadequate production of TH, and an inadequate iodine intake is the most common cause of hypothyroidism worldwide [4,42]. Hypothyroidism may be present at birth (congenital hypothyroidism) or arise in later life (acquired hypothyroidism). Criteria for the diagnosis of overt hypothyroidism are a high TSH and low T4, or normal T4 with a TSH greater than 10 mU/L; subclinical hypothyroidism is defined by high TSH and a normal T4. Symptoms of hypothyroidism include fatigue, cold intolerance, constipation, bradycardia, delayed reflexes, myxedema of the face or extremities, and goiter [4,42].

Sequelae of chronic hypothyroidism include other IDDs (Table 1). In particular, iodine deficiency and hypothyroidism during key childhood growth phases, and until the point at which bone maturation and epiphyseal closure occurs, can lead to reduced and/or delayed growth, reduced linear bone growth velocity, and delayed bone maturation [14,43]. In some cases, effects are irreversible, and can cause dwarfism, where the limbs are disproportionately short compared with the trunk.

Though each of the principal life stages demonstrates a critical need for iodine to support growth processes, evidence from across the literature is increasingly pointing towards the need for iodine sufficiency before a woman enters gestation [44,45,46,47]. Though much of the work in this area surrounds optimal neurodevelopment [44], it follows that growth processes may also be affected and it thus deserves mention here (Table 1).

In pregnancy, maternal iodine intakes and/or stores must be adequate to maintain a production of TH that is sufficient for both maternal and fetal needs. Yet, if iodine intakes before pregnancy are inadequate and iodine stores low, there is a risk that thyroid hormone synthesis will be insufficient for the needs of early pregnancy. The principal reasoning for this is the time needed to synthesize thyroid hormone after ingestion. Whilst iodine absorption from the gastrointestinal tract following ingestion is rapid [48], the synthesis of thyroid hormone and integration of iodine as iodinated tyrosines into stores may take up to several weeks [44,49]. Many women do not find out that they are pregnant until well within the first trimester, at which point supplement use may be too late to counteract the deleterious effects of a lack of iodine early in pregnancy [44,45,46,47]. Effects may even be seen in settings of mild-to-moderate iodine deficiency, which is today more often observed than severe deficiency, including in high-income countries with robust salt iodization schemes [50].

Finally, thyroid dysfunction has been associated with subfecundity, i.e., the ability to successfully conceive [51]. In a study in the US, a lower UIC (<50 μg/g creatinine) was associated with a 46% reduction in fecundity compared to women with UIC >100 μg/g creatinine [52]. In a large cohort study in Norway, iodine intakes of <100 μg/day were associated with increased subfecundity [47].

During pregnancy, the increased demand for iodine is potentiated by an increase in human chorionic gonadotropin (hCG) following oocyte fertilization, increased circulating estrogens that cause a progressive increase in thyroxine-binding globulin, and increased maternal renal excretion [53,54,55]. In early pregnancy, maternal T4 must cross the placenta until the fetus is able to organify iodide from the circulation at about week 16–20 [56]. Furthermore, the placenta likely protects the fetus from iodine deficiency. The expression of the sodium-iodide symporter (NIS) in placental syncytiotrophblast cells is upregulated by hCG [57]. As intracellular iodide increases in these cells, iodide is transported from the maternal to the fetal circulation [57]. The placenta may also function as a storage organ for fetal iodine supply [58]. The optimal function of these mechanisms is most likely under conditions of euthyroidism, decreasing in functionality as iodine (from dietary intake and liberation of maternal stores) decreases. In severe iodine deficiency, when intakes are low and maternal stores depleted, these mechanisms may not be able to adequately protect the fetus, thereby elevating the risk of suboptimal growth and delayed development.

In our systematic review [59], results of meta-analyses support these hypotheses. In a pooled result from six randomized and non-randomized controlled trials, no effect of iodine supplementation was found on birthweight, though when results were stratified by maternal iodine status, supplementation of severely iodine-deficient pregnant women led to a 200 g greater birthweight compared to controls, whereas in the moderate-to-mild deficient group, supplementation had no effect.

In Peru, severely-iodine deficient pregnant women treated with intramuscular iodized oil gave birth to infants 4.7 cm longer compared with women who received a placebo [60]. In Algeria, mothers treated with oral iodized oil had infants with a greater birthweight than untreated women [61]. In Bangladesh, maternal iodine intakes were positively associated with infant birthweight and length, though interestingly only in males, in whom the mean weight increased by 70 g and length by 0.41 cm, after a 500 μ/L increase in maternal UIC [62]. Spanish women with mildly insufficient iodine intakes in the third trimester were 13% less likely to have an infant small for gestational age than women with severe iodine deficiency [63], and in England, a trend for SGA or low-birthweight infants with iodine deficiency has been observed across several studies [64,65]. However, results are equivocal [66,67]. Our systematic review found no effect of iodine supplementation on infant length at birth [59]. In Papua New Guinea, when the children of mothers treated with iodized oil or placebo were followed at age 15, there was no difference in height [68]. This indicates confounding factors and the challenging nature of studies aiming to assess differences in somatic growth, and points to its multifactorial nature.

In early infancy, somatic growth is rapid, with an average of 25 cm length gained during the first 12 months [36]. Considering this, we might postulate that iodine deficiency has a greater impact on growth in infants and young children, when growth associated with TH is fastest [37]. Infants are at particular risk from inadequate intakes; neonatal iodine stores are scant [69,70] and a positive iodine balance is required from birth; infants require around 7 μg iodine/kg bodyweight at this time to replete thyroidal iodine stores in addition to daily iodine turnover [71]. During infancy, breastmilk iodine concentration (BMIC) determines the iodine status of exclusively breastfed infants, making sufficient iodine intakes also critical for lactating women [58].

A study in Morocco aptly demonstrates the importance of breastfeeding in promoting infant iodine repletion and healthy thyroid function [72]. In regions of moderate-to-severe iodine deficiency in Morocco, lactating women receiving 400 mg of oral iodized oil were able to provide sufficient iodine to their breastfed infants to achieve euthyroidism by 3 months of age, whereas direct supplementation to infants was less effective. The groups showed no difference in growth parameters, however. Yet, in Iranian infants aged 6 months, breast milk iodine concentration was positively associated with weight-for-length z-scores [73]. In China, the lowest mean values of height-for-age z-scores and weight-for-age z-scores in infants aged under 6 months were found when maternal UIC was well below requirements [74]. Furthermore, models calibrated on data from US lactating women predicted that a moderate iodine deficiency in these women would result in a ~30% decrease in T4 in their infants [75], underlining the critical role breast milk or properly iodized breast-milk-substitutes have on assuring iodine intakes in young infants.

Findings from a longitudinal study demonstrate the importance of IGF-1 on infant growth [76]. Wiley et al. followed an Indian birth cohort of n = 122 infants at age two years, revealing a positive association between infant length and IGF-1 [76]. The authors did not measure the iodine status or thyroid function of the study population; however, further work in the same area of India suggested adequate iodine intakes in that population [77].

Studies in school-age children have demonstrated the relationship between iodine deficiency [78], or iodine repletion [79], and their influence on indirect biomarkers of growth, such as IGF-1 and their binding proteins. Pubertal children from a severely iodine-deficient area of Turkey had significantly lower IGF-1 and IGF-binding protein-3 (IGFBP-3) levels than counterparts living in an area of mild deficiency [80]. When iodized salt was introduced in a severely iodine-deficient area of Morocco, median IGF-1 concentrations improved in the 7–10-year-old school children living in households using the iodized salt [79,81]. The iodine repletion of school-age children using iodized oil in areas of moderate or mild iodine deficiency increased serum IGF-1 and IGFBP-3 compared to a placebo [82,83].

#### 2.3.2. Excessive Iodine Intakes

There is less evidence that excessive iodine intakes may have sequelae on growth processes or outcomes, since physiological mechanisms exist that largely prevent adverse effects from excessive iodine exposure. The healthy thyroid is a highly flexible organ, capable of adapting to various levels of iodine. The WHO maximal tolerated upper level of iodine, safe for healthy, euthyroid persons is 1 mg iodine per day, equivalent to 17 μg/kg body weight [17], though most euthyroid adults, without underlying thyroid disease and living in iodine-sufficient areas, can tolerate a chronic excess iodine intake of up to 2 g/day without a clinical effect [84].

Following exposure to excessive iodine intake, the Wolff–Chaikoff effect protects the organism from excessive TH production. This mechanism is effectively a block in TH synthesis when plasma iodide concentrations exceed a critical level, and was elaborated by Wolff and Chaikoff in 1948 [85]. Once the plasma iodide concentration falls again, an “escape” from this block occurs. However, prolonged high iodine intakes may prevent an escape; indeed, this is the mechanism by which iodine is used prophylactically to protect the thyroid gland in the case of a nuclear accident [86]. However, unintended exposure to iodine intakes beyond the critical level can lead to hypothyroidism and/or goiter in vulnerable individuals, particularly those with an underlying thyroid disorder. Effects usually disappear following a decrease in iodine intake [87].

This prompts the question: is a temporary cessation in the formation of TH deleterious during periods of intense growth, such as pregnancy or infancy? Though capable of iodide organification from gestational weeks 16 to 20, the developing fetal thyroid cannot escape from the Wolff–Chaikoff effect until about week 36 weeks [88]. Sequelae due to maternal–fetal excess iodine exposure include, in severe cases, the adverse formation of the thyroid gland during embryogenesis [89], fetal goiter that can obstruct the neonatal airway at delivery [90,91], or congenital hypothyroidism [88,92]. Even mildly excessive intakes may cause maternal hypothyroidism or isolated hypothyroxinaemia, thus reducing the availability of TH for growth processes. Iodine supplementation that is only started early in pregnancy in women having a low iodine intake and low stores periconceptually may also have the same effect [46]. Iodine-induced hypothyroid states may have the same effect on growth as hypothyroidism caused by iodine insufficiency (Part 2.c.i.). In some cases, the effects are reversible: maternal consumption of seaweed soup during and after pregnancy led to hypothyroidism in the infant; however, the interruption of breastfeeding and short-term therapy with synthetic TH (levothyroxine) corrected thyroid function and the infant had a normalized growth trajectory [93].

Excessive iodine intake increases the risk of subclinical hypothyroidism, where TSH is elevated yet T4 is normal. In an area of China exposed to high iodine concentrations in the drinking water, children aged 7 to 13 years showed a greater prevalence of subclinical hypothyroidism and other thyroid disorders compared with similar children living in an area with sufficient but not excessive iodine intakes (control children) [94]. Interestingly, however, the high-iodine children were, on average, 4 cm taller than the control children (*p* = 0.001). This example perhaps illustrates the importance of iodine for growth, yet shows the delicate balance between low, optimal and elevated iodine intakes. It has previously been demonstrated in several life-stage groups, that an optimal iodine balance exists in a U-shaped curve [95,96,97,98]. Most cases of subclinical hypothyroidism revert to normal; however, between 1% and 5% of individuals will develop overt disease [99].

#### 2.3.3. Hyperthyroidism, Thyrotoxicosis, and Graves’ Disease

Unlike the link between hypothyroidism and an insufficient iodine intake, hyperthyroidism is usually due to autoimmunity rather than an excessive iodine intake. Hyperthyroidism is defined by elevated circulating THs: elevated T4 accompanied by a low or undetectable TSH. A subclinical diagnosis refers to a low TSH with normal T4 levels. Thyrotoxicosis refers to the clinical picture of excess circulating THs, irrespective of the source [100]. Finally, Graves’ disease is an autoimmune disorder and the most common cause of hyperthyroidism [100,101,102] Presenting symptoms of hyperthyroidism, including in children, include warm moist skin, diarrhea, tachycardia or cardiac arrhythmia, asthma or respiratory disease [100,102]. Goiter may be present due to the lymphocytic infiltration of the thyroid gland [42], and there may be unexpected weight loss or failure to gain weight, e.g., in childhood [103,104].

Graves’ disease during pregnancy is associated with pregnancy loss and stillbirth, prematurity, low birth weight, and intrauterine growth restriction, as well as maternal complications such as hypertension and congestive heart failure. Risks are higher when the disease is uncontrolled, but persist even if under control [105,106]. Infants of mothers with Graves’ disease may have a higher risk of neonatal thyrotoxicosis at birth, which can lead to significant morbidity and mortality if not treated [107].

If hyperthyroidism or Graves’ disease in childhood is untreated, it can lead to negative growth sequalae. Accelerated linear growth and advanced bone age caused by hyperthyroidism [40,42,103] can cause the child to not attain full height potential due to the premature closure of the end of long bones (epiphyses) or premature closure of cranial plates [38,40]. In severe cases, craniosynostosis may result from premature fusion of the skull sutures, which may also result in cognitive deficits [5]. If hyperthyroidism remains uncorrected, calcification and bone formation is increased, alongside enhanced bone resorption [39,108]. Bone mineral density is thus weakened with age [38], with increased porosity and decreased cortical thickness in cortical bone [39].

## 3. Other Micronutrients for Consideration

This review has described the effects that iodine may have on growth, via its critical role in thyroid function, with optimal thyroid function being the true facilitator for optimized growth trajectories. Nutrients rarely function alone in the human body, and it is therefore also important to note the other micronutrients crucial for optimal thyroid function.

Iron is a requirement for the heme-dependent thyroid peroxidase enzyme, that catalyzes the oxidation of iodide and the binding of iodine to the tyrosyl residues of thyroglobulin [109]. Selenium is critical for the selenoprotein iodothyronine deiodinase enzymes that catalyze the removal of iodide from T4 in the conversion of T4 to T3. Selenoproteins also have an important antioxidant role in the thyroid gland [110]. Zinc regulates both the synthesis and mechanism of action of THs. Zinc acts as a connector between T3 and its nuclear receptor in the hypothalamus to stimulate thyrotropin-releasing hormone synthesis, which, in turn, stimulates the synthesis of TSH and thereby regulates TH synthesis. Zinc is also involved in the activity of deiodinase selenoprotein enzymes and can modify the structures of the transcription factors involved in the synthesis of THs [111]. Vitamin A has an intricate and complex relationship with thyroid function and vitamin A status can influence iodine uptake by the thyroid, which is lessened in vitamin A deficiency. Vitamin A deficiency also impairs the synthesis of thyroglobulin, the glycoprotein structure on which THs are formed [112,113,114]. Finally, vitamin D, already associated with growth through its role in regulating bone metabolism and calcium and phosphorus homeostasis, may have other impacts on growth via its association with the GH-IGF-1 axis [115], which itself is associated with thyroid hormone, as reviewed above. Furthermore, through its significant role in immune system modulation, vitamin D may be implicated in the development of autoimmune thyroid diseases such as Hashimoto’s thyroiditis and Graves’ disease; however, more research is required to substantiate this hypothesis [116].

## 4. Discussion

With the development of Universal Salt Iodization (USI) programs worldwide, there has been significant progress in reducing iodine deficiency disorders in recent decades. At the time of writing, the Iodine Global Network recorded 26 countries with a risk of insufficient iodine intakes, based on data from school-age children [24]. However, the parity of iodized salt programs within countries remains an issue [117], possibly putting part of the population at risk, particularly vulnerable groups such as pregnant and lactating women and young children, in whom somatic growth, and thus the requirement for iodine, is highest. Public health policy should continue to emphasize USI policy, or the iodization of salt in staple foods or other staple condiments, and assure adequate monitoring and surveillance, including sentinel surveys in vulnerable population groups to inform where iodized salt programs may not be as effective as assumed [118,119,120]. Since, if mild processing disorders linked to iodine deficiency may be increasing as reported, it follows that growth may too be affected.

On the other hand, 13 countries recorded a prevalence of excessive intakes, i.e., intakes higher than those required to prevent iodine deficiency [6]. Excessive intakes may be due to over-iodization or poor monitoring of USI, additive dietary factors, e.g., seaweed consumption, use of dietary supplements, or arising from environmental situations such as groundwater with a high iodine concentration [121]. Though perhaps not as critical as insufficient iodine intakes, as reviewed above, excessive intakes may also have negative long-term effects on growth.

This review has described several human studies reporting growth data in conjunction with iodine intakes, and underlined the public health importance of assuring sufficient population iodine intakes throughout life, not just at periods associated with rapid growth such as pregnancy and infancy. These studies have used several anthropometric and biochemical markers to describe growth or growth changes, including height or length, birth outcomes such as birth weight, length or SGA, z-scores of height, weight and age, and circulating concentrations of IGF and IGFBP-3. Further potential indicators of growth include head circumference, body mass index, mid-upper arm circumference, and child growth rate. The indicators chosen will depend upon the study design. Several other micronutrients are also critical to consider when assessing the relationship of iodine to growth, including iron, selenium, zinc, and vitamins A and D, and studies should therefore also consider status of these micronutrients in the sample population.

## Figures and Tables

**Table 1:** Iodine deficiency disorders.

Life Stage Group	Health Consequences of Iodine Deficiency	
Fetus	Failed pregnancy Stillbirth Congenital anomalies Perinatal mortality	Goiter Hypothyroidism Increased susceptibility to nuclear radiation
Neonate	Endemic congenital hypothyroidism Infant mortality	
Child, Adolescent	Impaired mental function Delayed physical development Iodine-induced hyperthyroidism	
Adult	Impaired mental function Iodine-induced hyperthyroidism	
Neonate	Failed pregnancy Stillbirth Congenital anomalies Perinatal mortality	

Adapted from [6].

**Table 2:** Iodine intake recommendations during growth life stages.

	European Union Scientific Committee on Foods (2014, 2006)		US Institute of Medicine (2001)		World Health Organization (2004)	
	AI	TUL	AI	TUL	RI	TUL
	µg/Day		µg/Day		µg/Day	
Periconception	150	500	150 ^c^	1100	150 (adolescents and adults >13 y)	30 ^a^ (adolescents and adults >13 y)
Pregnant women	200	600	220	1100	250	40 ^a^
Lactating women	200	600	290	1100	250	40 ^a^
Infants and young children						
Individual level iodine intake recommendations						
0–6 mo ^1^	ND	ND	110	ND	15 ^a^	150 ^a^
7–12 mo ^1^	70	ND	130	ND	15 ^a^	140 ^a^
1–3 y ^1^	90	200	65 ^b^/90 ^c^	200	6 ^a^ (2–5 y)	50 ^a^
Population iodine intake recommendation						
0–59 months ^1^					90	180
Older children and adolescents						
4–6 y	90	250	90 ^c^ (4–8 y)	300 (4–8 y)	6 ^a^ (2–5 y)	50 ^a^
7–10 y	90	300	120 ^c^ (9–13 y)	600 (9–13 y)	4 ^a^ (6–12 y)	50 ^a^ (7–12 y)
11–14 y	120	450				
15–17 y	130	500	150 ^c^ (14–18 y)	900 (14–18 y)	150 (adolescents and adults >13 y)	30 ^a^ (adolescents and adults >13 y)

^1^ Recommendations are age-relative and are not per source of iodine (i.e., breast milk, formula, complementary food) nor are based on whether the child is weaning or has already been weaned. Abbreviations: AI; adequate intake; mo, months; ND, not defined; RDA, recommended daily allowance; RI, recommended intake; TUL, tolerable upper level; y, years. ^a^ recommendation per kg bodyweight, ^b^ EAR, estimated average requirement; ^c^ RDA, recommended daily allowance. Sources: [17,18,19,20].

## Data Availability

Not appliable.
